# Optimal combination periprosthetic vasculature visualization and metal artifact reduction by spectral computed tomography using virtual monoenergetic images in total hip arthroplasty

**DOI:** 10.1186/s13244-023-01533-3

**Published:** 2023-10-26

**Authors:** Jie Zhao, Qiang Cheng, Chuan Liu, Qiqi Wang, Yuchan Lv, Ziyi Tang, Yuxi Luo, Haitao Yang

**Affiliations:** 1 Department of Radiology, The First Affiliated Hospital of Chongqing Medical University, 1 Youyi Road, Yuzhong District, Chongqing, 400016 China; 2 Department of Orthopedics, The First Affiliated Hospital of Chongqing Medical University, Chongqing, China

**Keywords:** Energy spectrum CT, Virtual monoenergetic imaging, Metal artifact reduction, Periprosthetic vasculature

## Abstract

**Objectives:**

To investigate the optimal parameters of spectral CT for preferably visualizing the periprosthetic vasculature and metal artifact reduction (MAR) in total hip arthroplasty (THA).

**Methods:**

A total of 34 THA of 30 patients were retrospectively included. Image reconstructions included conventional image (CI), CI combined with MAR (CI_MAR_), and virtual monoenergetic images (VMI) combined with MAR (VMI_MAR_) at 50–120 keV. The attenuation and standard deviation of the vessel and artifact, and the width of artifact were measured. Qualitative scoring was evaluated including the vascular contour, the extent of artifact, and overall diagnostic evaluation.

**Results:**

The attenuation, noise of the vessel and artifact, and the width of artifact decreased as the energy level increased (*p* < 0.001). The downtrend was relatively flat at 80–120 keV, and the vascular attenuation dropped to 200 HU at 90 keV. The qualitative rating of vascular contour was significantly higher at CI_MAR_ (3.47) and VMI_MAR_ 60–80 keV (2.82–3.65) compared with CI (2.03) (*p* ≤ 0.029), and the highest score occurred at 70 and 80 keV (3.65 and 3.56). The score of the extent of artifact was higher at VMI_MAR_ 80 keV than CI_MAR_ (3.53 VS 3.12, *p* = 0.003). The score of the overall diagnostic evaluation was higher at VMI_MAR_ 70 and 80 keV (3.32 and 3.53, respectively) than CI_MAR_ (3.12) (*p* ≤ 0.035).

**Conclusion:**

Eighty kiloelectron volts on VMI_MAR_, providing satisfactorily reduced metal artifacts and improved vascular visualization, can be an optimal recommended parameter of spectrum CT for the assessment of periprosthetic vasculature in THA patients.

**Critical relevance statement:**

The metal artifact is gradually reducing with increasing energy level; however, the vascular visualization is worsening. The vascular visualization is terrible above 100 keV, while the vessel is disturbed by artifacts below 70 keV. The best performance is found at 80 keV.

**Key points:**

• VMI_MAR_ can provide both reduced metal artifacts and improved vascular visualization.

• Eighty kiloelectron volts on VMI_MAR_ performs best in vascular visualization of total hip arthroplasty patients.

• Energy spectrum CT is recommended for routine use in patients with total hip arthroplasty.

**Graphical Abstract:**

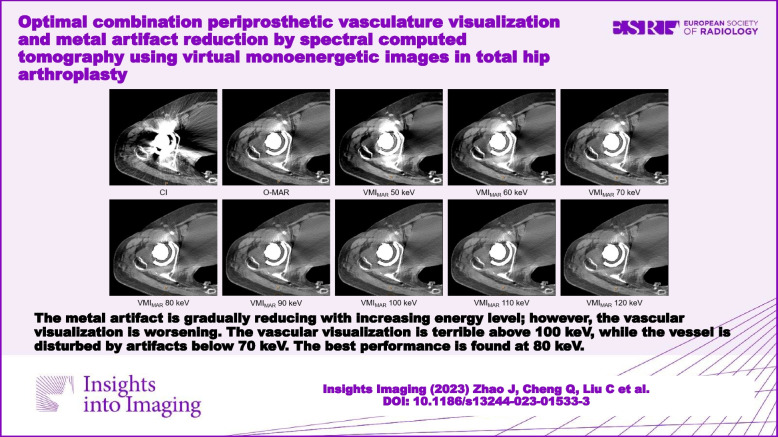

## Background

Total hip arthroplasty (THA) has become one of the most cost-effective and successful orthopedics procedures in patients with severely symptomatic hip disease [1]. With a significant increase in annual rates of primary and revision THA, accompanied by increasing concern about intraoperative and postoperative complications [2, 3]. Vascular injuries are uncommon but potentially devastating during replacement. The external iliac and common femoral vessels at the periprosthetic area are the most injured [4]. Early diagnosis and intervention are critical to avoid these dreaded complications. In addition, THA has been reported as an increased risk of peripheral arterial disease (PAD), and patients with THA exhibited a 1.24-fold higher risk of developing PAD than patients without THA [5–9]. Therefore, accurate assessment of relevant anatomy and pathology of periprosthetic vessels is essential to THA patients in both preoperative planning and postoperative follow-up for avoiding vascular-related complications.

Computed tomographic angiography (CTA) has gained wide acceptance as a valid modality for the noninvasive assessment of peripheral vasculatures. However, the metal artifacts resulting from beam hardening, photon starvation, and scattering are a great challenge for visualizing the periprosthetic vessels in THA patients [10–12]. Energy spectral CT has emerged as a promising tool in diagnostic imaging with multiple potential clinical applications. Virtual monoenergetic images (VMI) and orthopedic metal artifact reduction (O-MAR) software based on DE-CTA can provide high image quality of vessels and remarkably reduce metal artifacts compared to conventional CT and CTA, respectively [13–20]. Nevertheless, it is still unknown whether these advanced techniques are suitable for the evaluation of periprosthetic vessels in THA patients. According to previous studies, the recommended parameters seem somewhat contradictory, high keV level images prefer to display MAR while low keV levels are better for vessels, between optimal visualization of the vessel and MAR of the prosthetic joint [14, 21–23]. And the impact of O-MAR techniques on the surrounding vasculature is also unclear.

Thus, this study aimed to assess the quantitative image quality of VMI of DE-CTA and to define the optimal parameters for better visualizing the periprosthetic vasculature combined with metal artifact reduction in THA patients.

## Materials and methods

### Patients

This study was approved by our local institutional review board. Informed consent was waived because of its retrospective character. We included DE-CTA examinations of 34 THA joints with 30 patients (19 men, 11 women, mean age 66.13 ± 12.77 years, range 32–87 years, BMI 16.9–29.4) in our institution. The inclusion criteria were (1) patients were performed a preoperative evaluation of revision THA and (2) patients were conducted within the routine clinical practice for the evaluation of peripheral artery after THA. The exclusion criteria were examinations with severe motion artifacts, images with poor quality, or incomplete raw data influencing spectral reconstruction.

### Image acquisition

All scans were performed on a dual-layer detector CT scanner (IQon spectral CT, Philips Healthcare). The scanning parameters were as follows: tube voltage, 120 kVp; collimation, 64 × 0.625; pitch, 0.391; rotation time: 0.5 s; matrix, 512 × 512. A bodyweight adapted bolus of contrast agent (370 mg I/mL, Iopromide, Ultravist, Bayer Healthcare, Germany) was injected through a peripheral vein with a flow rate of 2.5–4.0 mL/s. Bolus-tracking technique with a delay of 35–40 s was used for contrast-enhanced imaging.

### Image reconstruction

Conventional image, conventional image combined with O-MAR, and VMI combined with O-MAR were reconstructed, which would be referred to as CI, CI_MAR_, and VMI_MAR_. CI, CI_MAR_, and VMI_MAR_ were reconstructed in the axial plane with a slice thickness of 0.8 mm and section increments of 0.6 mm for meeting the clinical pre-operative requirement of 3D printing customized prostheses. CI, CI_MAR_, and VMI_MAR_ were computed using a dedicated reconstruction algorithm (Spectral B, level 3, Philips Healthcare). To ensure sufficient opacification of the vessel (> 200 HU), the VMI_MAR_ was reconstructed in the range of 50 to 120 keV at 10-keV intervals as vascular opacification is insufficient over 120 keV levels [13, 24].

### Image analysis

Quantitative and qualitative analysis was carried out using the proprietary image viewer (Philips IntelliSpace Portal).

**Fig. 1:**
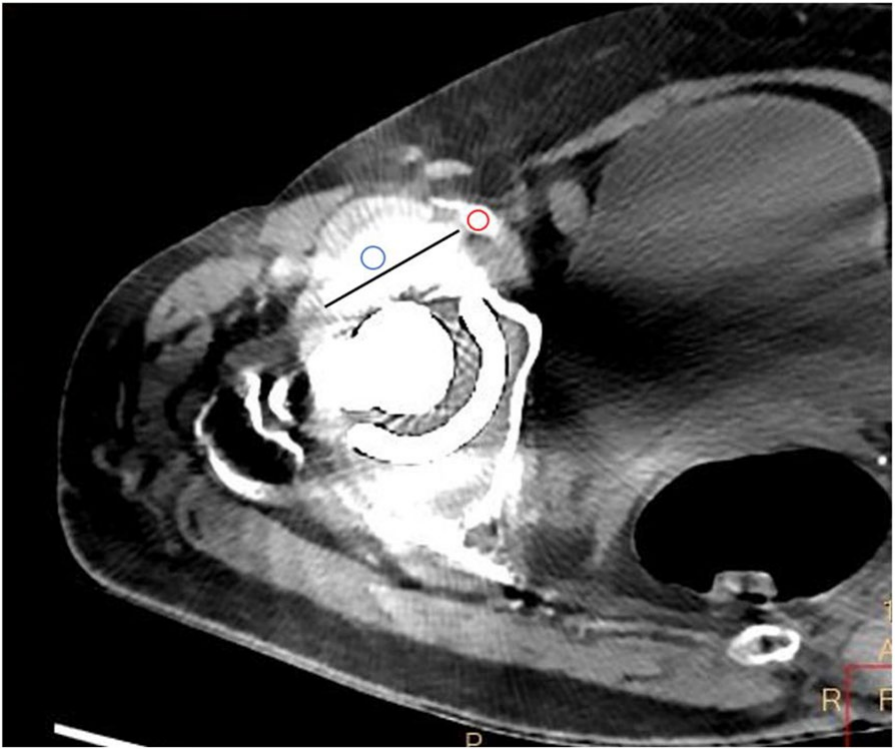
Illustration of the quantitative assessment of image quality. The axial slices where the vessel was most disturbed by the metal artifact were selected. Regions of interest (ROI) were placed in the external iliac or common femoral vessels (red circle) and the most pronounced hyperdense artifact (blue circle). Attenuation values and standard deviations within the ROI were automatically recorded. Additionally, the width of the most pronounced hyperdense artifact was measured close to the metal implant in its largest diameter (black line). ROIs were kept constant and measurements of width were conducted in the same location on CI, CI_MAR_, and VMI_MAR_ (CI indicates conventional images; CI_MAR_ indicates conventional images combined with orthopedic metal artifact reduction; VMI_MAR_ indicates virtual monoenergetic images combined with orthopedic metal artifact reduction)

For quantitative assessment, the axial slices where the vessel was most disturbed by the metal artifact were selected. The regions of interest (ROI) were placed in the external iliac or common femoral arteries and the most pronounced hyperdense artifact close to the vessel by one musculoskeletal radiologist (Zhao, with 2 years of clinical experience). ROIs were placed on CI with soft tissue window (width 360 HU, center 60 HU) [17, 25]. The location and size of the ROIs (contour of artery and 20 mm^2^ for metal artifacts) were then used as a template for the other image reconstructions and thus kept constant on CI, CI_MAR_, and VMI_MAR_ [17, 25, 26]. Attenuation values and standard deviation within the ROI were automatically recorded from the same ROI on CI, CI_MAR_, and VMI_MAR_ at 50 keV, 60 keV, 70 keV, 80 keV, 90 keV, 100 keV, 110 keV, and 120 keV. In addition, the width of the most pronounced hyperdense artifact was manually measured (Fig. 1).

For qualitative assessment, the vascular contour, the extent of artifact, and overall diagnostic evaluation were separately assessed by two musculoskeletal radiologists (Zhao and Yang, with 2 and 18 years of clinical experience, respectively) using a five-point Likert scale in the same axial slice. The radiologists had accepted a series of training about the subjective scoring system and performed several group meetings for the consensus evaluation referring to previous studies [20, 27, 28]. The vascular contour was graded as follows: blurred vascular contour = 1, less clear vascular contour = 2, basically sharp vascular contour = 3, relatively sharp vascular contour = 4, and very sharp vascular contour = 5 [27]. The extent of artifact was rated as follows: massive artifacts = 1, pronounced artifacts = 2, moderate artifacts = 3, little artifacts = 4, no artifacts = 5. The overall diagnostic evaluation was rated as follows: nondiagnostic = 1, poor quality = 2, moderate quality = 3, good quality = 4, and excellent quality = 5 [28].

### Statistical analysis

Statistical analysis was performed using SPSS software version 26 (IBM Corp., Armonk, NY, USA). All data were expressed as mean ± SD. Quantitative measurements (attenuation, noise of the vessel and artifact, and the width of artifact) were tested for normal distribution using the Shapiro–Wilk test, and repeated measures analysis of variance was applied if the data had a normal distribution; otherwise, the Friedman test and Wilcoxon test were used to compare the variables among the reconstructions. And the Friedman test and Wilcoxon test were also used for qualitative measurements. A *p*-value of less than 0.05 was considered statistically significant and adjustment for multiple comparisons was implemented with Bonferroni correction. Inter-rater agreement was assessed using Cohen’s kappa analysis.

## Results

### Reduction of hyperdense artifacts

**Fig. 2:**
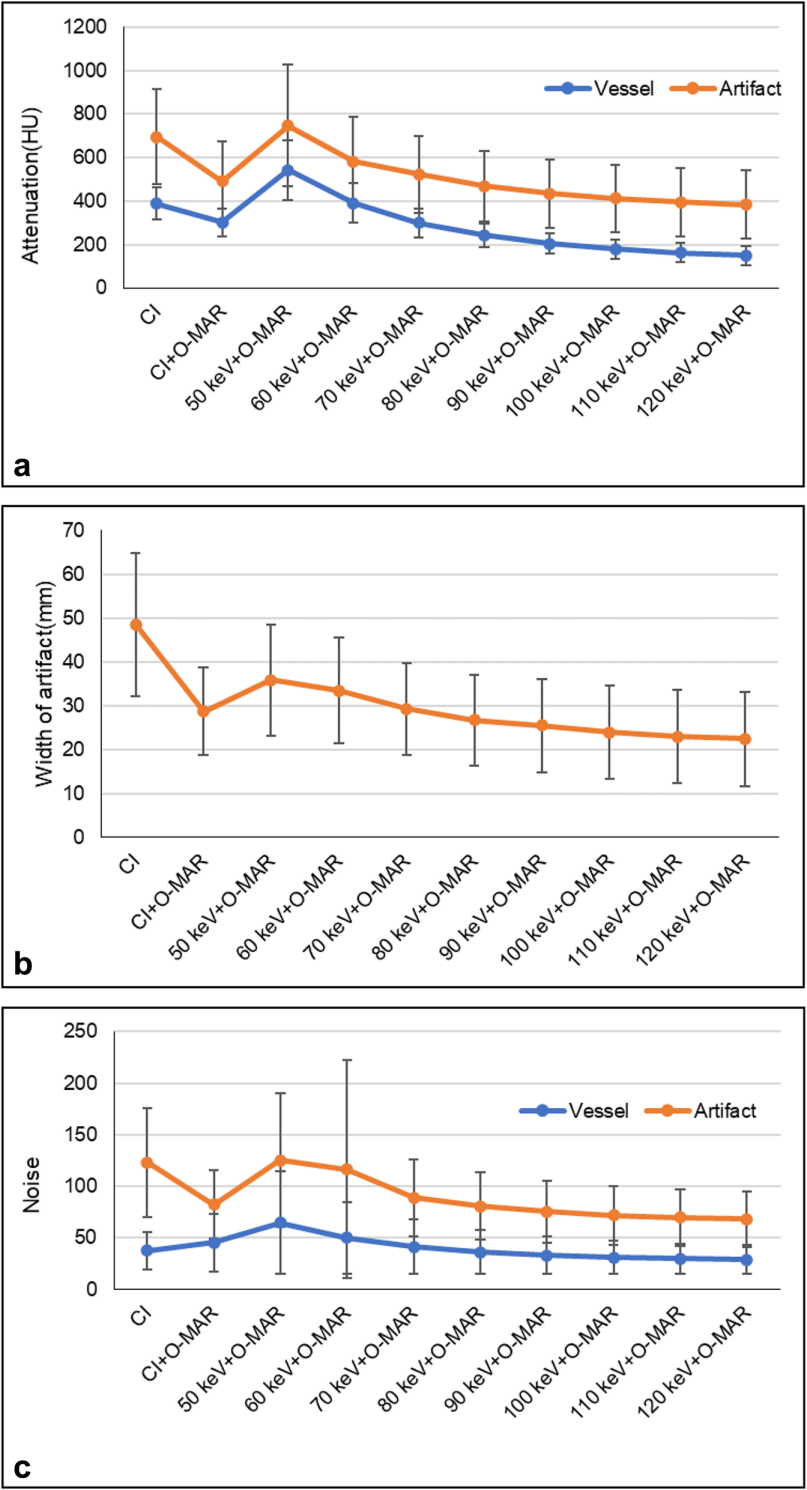
The trend chart of the attenuation (**a**), the noise (**c**) of the vessel and artifact, and the width of artifact (**b**) in CI, CI_MAR_, and VMI_MAR_ of various keV levels (50 – 120 keV). The attenuations, widths, and noises of hyperdense artifacts and periprosthetic vessels all gradually decrease with increased keV levels. The similar downtrends all show sharp at 50–70 keV and, relatively flat at 80–120 keV. At 80 keV level, the vascular attenuation is higher than 200 HU which is sufficient vascular opacification, simultaneously, the attenuation of artifacts is also below 500 HU (**a**). The vascular attenuation gradually drops to 200 HU at 90 keV. And the change of the width of artifacts, the noises of vessels and artifacts tend to be stable and small among the keV levels over 80 compared to the 50–70 keV levels (**b**–**c**). The vascular noise on VMI_MAR_ at 50–70 keV was even higher than that on CI (**c**)

Compared to the positive Hounsfield Unit (HU) of hyperdense artifacts measured on CI (696.04 ± 218.17 HU), the attenuation of artifacts on CI_MAR_ significantly decreased (490.82 ± 181.42 HU, *p* < 0.001); the attenuation of artifacts on the VMI_MAR_ at 60 – 120 keV were lower than CI, and the differences were significant on the VMI_MAR_ at 80–120 keV (*p* < 0.001) (Table 1). And the significant difference was detected among the different keV levels of the VMI_MAR_ (*p* < 0.05). In the VMI_MAR_ series, the attenuation of hyperdense artifacts gradually decreased with increased keV levels; the attenuation of artifacts on VMI_MAR_ 80 keV was significantly lower than CI_MAR_ (469.36 ± 162.21 HU VS 490.82 ± 181.42 HU, *p* = 0.005). The downtrend showed sharp at 50–70 keV and, relatively flat at 80–120 keV (Fig. 2A).

**Table 1:** Comparisons of the attenuation and noise of vessel and artifact

	**CI**	**CI**_**MAR**_	** VMI**_**MAR**_							
			**50 keV**	**60 keV**	**70 keV**	**80 keV**	**90 keV**	**100 keV**	**110 keV**	**120 keV**
Attenuation (HU)										
Vessel	387.26 ± 73.65	302.17 ± 62.81	542.52 ± 136.87	390.50 ± 90.73	299.15 ± 66.07	242.06 ± 53.50	204.77 ± 47.69	179.41 ± 45.25	161.55 ± 44.37	148.59 ± 44.28
Artifact	696.04 ± 218.17	490.82 ± 181.42	747.31 ± 281.64	581.37 ± 204.93	522.28 ± 174.86	469.36 ± 162.21	434.85 ± 158.17	411.86 ± 156.53	395.36 ± 157.14	383.38 ± 158.12
Noise										
Vessel	37.42 ± 18.57	45.34 ± 27.78	64.61 ± 50.01	49.83 ± 34.86	41.36 ± 26.08	36.31 ± 20.97	33.20 ± 17.92	31.12 ± 15.99	29.79 ± 14.81	28.86 ± 14.01
Artifact	122.92 ± 53.34	82.08 ± 33.07	125.37 ± 64.79	116.57 ± 105.37	88.76 ± 37.15	80.68 ± 32.42	75.55 ± 29.99	72.0 ± 28.51	69.70 ± 27.75	68.08 ± 27.28
Width of artifact (mm)	48.51 ± 16.33	28.74 ± 9.97	35.93 ± 12.68	33.47 ± 12.11	29.25 ± 10.49	26.76 ± 10.37	25.53 ± 10.59	23.89 ± 10.61	22.91 ± 10.63	22.41 ± 10.69

*CI *conventional image, *CI*_*MAR*_ conventional image combined with orthopedic metal artifact reduction, *VMI*_*MAR*_ virtual monoenergetic images combined with orthopedic metal artifact reduction

The average width of the hyperdense artifacts on CI was 48.51 ± 16.33 mm, and significantly reduced on CI_MAR_ (28.74 ± 9.9 mm, *p* < 0.001); the average width of the hyperdense artifacts on VMI_MAR_ was lower than CI, and the differences were significant on the VMI_MAR_ at 70–120 keV (*p* < 0.001, Table 1). The average width of the hyperdense artifacts gradually decreased with increased keV levels and was significantly lower than CI_MAR_ on VMI_MAR_ 80 keV (26.76 ± 10.37 mm VS 28.74 ± 9.9 mm, *p* < 0.001), with the lowest occurring on VMI_MAR_ 120 keV (22.41 ± 10.69 mm, *p* < 0.001). A similar downtrend was observed at the different keV levels. The difference between 50 and 80 keV was about 9 mm, while it was only about 3 mm between 90 and 120 keV (Fig. 2B).

### Vascular attenuation

Vascular attenuation was found highest on VMI_MAR_ 50 keV (555.19 ± 141.19 HU) compared to all other reconstructions. Compared to the vascular attenuation measured on the CI (387.26 ± 73.65 HU), the attenuation decreased on CI_MAR_ and VMI_MAR_ at 70–120 keV, and the differences were significant on CI_MAR_ and VMI_MAR_ at 80–120 keV (*p* < 0.001). In the VMI_MAR_ series, vascular attenuation gradually dropped to 200 HU at 90 keV with increased keV levels (Fig. 2A).

### Image noise in vessels and artifacts

Image noise in artifacts was found highest on CI and VMI_MAR_ 50 keV (122.92 ± 53.34, 125.37 ± 64.79, respectively). In the VMI_MAR_ series, image noise in artifacts gradually decreased with increased keV levels. The downtrend was also steep at 50 – 80 keV and, relatively smooth at 90–120 keV. And no significant difference was detected at 100–120 keV (*p* > 0.655). Image noise in artifacts on VMI_MAR_ 80 keV was lower than CI_MAR_ without significant difference (80.68 ± 32.42 VS 82.08 ± 33.07, *p* = 0.274) (Table 1, Fig. 2C).

The image noise of the vessel was highest on VMI_MAR_ 50 keV (64.61 ± 50.01), then gradually decreased with the increased keV levels. The vascular noise on CI_MAR_ and VMI_MAR_ at 50–70 keV was even higher than that on CI (37.42 ± 18.57). Compared to vascular noise on CI_MAR_ (45.34 ± 27.78), vascular noise was lower on VMI_MAR_ at 70–120 keV, and the differences were significant on VMI_MAR_ at 80–120 keV (*p* = 0.004). In the VMI_MAR_ series, the downtrend was not obvious at 90–120 keV and without significant difference (*p* > 0.815) (Table 1, Fig. 2C).

### Reliability and subjective scoring

**Fig. 3:**
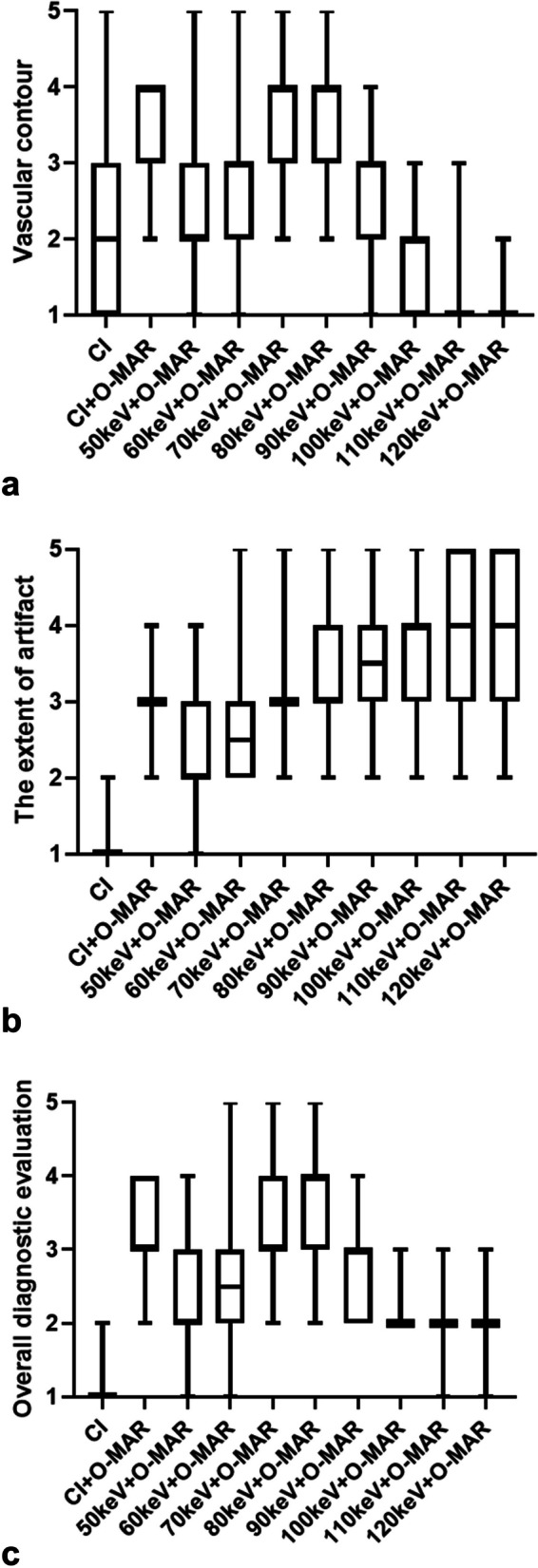
Comparisons of qualitative rating of vascular contour (**a**), the extent of artifact (**b**), and overall diagnostic evaluation (**c**) in CI, CI_MAR_, and VMI_MAR_ of various keV levels (50 – 120 keV). **a** In all reconstructions, the scores of vascular contours gradually increase to the highest level of 3.63 at 70 keV and 3.56 at 80 keV and then decrease to 1.09 at 120 keV. **b** The score of the extent of the artifact is 1.06 in CI, 3.12 in CI_MAR_ and increases to a maximum of 4.03 at 120 keV. And the score of the extent of the artifact at 80 keV is higher than CI_MAR_. **c** The scores of overall diagnostic evaluations gradually increase from the minimum (CI, 1.06) to 3.53 at 80 keV and then decrease to 1.91 in all reconstructions

The two reading radiologists had similar scores in 916 out of 1020 ratings (89.8%), with a kappa value of 0.865 (SE = 0.013, 95% CI: 0.840–0.890). The score of vascular contours was measured as 2.03 on CI and 3.47 on CI_MAR_. The score of vascular contours was higher on CI_MAR_ and VMI_MAR_ at 50–90 keV than on CI, and the differences were significant on CI_MAR_ and VMI_MAR_ at 60–80 keV (*p* ≤ 0.029). In the VMI_MAR_ series, the score of vascular contours gradually improved with the increased keV levels, higher than CI_MAR_ at 70 keV and 80 keV (3.65 and 3.56 respectively, *p* > 0.083), and then gradually decreased to the lowest at 120 keV (1.09). The score of the extent of artifact was measured as 1.06 on CI and 3.12 on CI_MAR_. The score of the extent of artifact was significantly higher on CI_MAR_ and VMI_MAR_ than on CI (*p* < 0.018). In the VMI_MAR_ series, score of the extent of artifact gradually improved with the increased keV levels, and was significantly higher on VMI_MAR_ 80 keV than on CI_MAR_ (3.53 vs. 3.12, *p* = 0.003), reaching the highest at 120 keV (4.03). However, no significant difference was detected at 80–120 keV (*p* > 0.291). The score of the overall diagnostic evaluation was measured as 1.06 on CI and 3.12 on CI_MAR_. The score of the overall diagnostic evaluation was significantly higher on CI_MAR_ and VMI_MAR_ than on CI (*p* < 0.043). In the VMI_MAR_ series, the score of overall diagnostic evaluation gradually progressed with the increased keV levels, higher than CI_MAR_ at 70 and 80 keV (3.32 and 3.53 respectively, *p* ≤ 0.035), and then gradually decreased to the lowest at 120 keV (1.91) (Table 2, Fig. 3).

**Table 2:** Comparisons of the qualitative image rating of all reconstructions

	**CI**	**CI**_**MAR**_	** VMI**_**MAR**_							
			**50 keV**	**60 keV**	**70 keV**	**80 keV**	**90 keV**	**100 keV**	**110 keV**	**120 keV**
Vascular contour	2.03 ± 0.97	3.47 ± 0.66	2.32 ± 1.07	2.82 ± 0.97	3.65 ± 0.65	3.56 ± 0.71	2.71 ± 0.72	1.82 ± 0.63	1.24 ± 0.50	1.09 ± 0.29
Extent of artifact	1.06 ± 0.24	3.12 ± 0.54	2.18 ± 0.76	2.62 ± 0.74	3.06 ± 0.65	3.38 ± 0.70	3.53 ± 0.75	3.85 ± 0.78	4.0 ± 0.82	4.03 ± 0.83
Overall diagnostic evaluation	1.06 ± 0.24	3.12 ± 0.64	2.12 ± 0.73	2.56 ± 0.75	3.32 ± 0.68	3.53 ± 0.66	2.88 ± 0.64	2.18 ± 0.39	2.00 ± 0.43	1.91 ± 0.38

*CI* conventional image, *CI*_*MAR*_ conventional image combined with orthopedic metal artifact reduction, *VMI*_*MAR*_ virtual monoenergetic images combined with orthopedic metal artifact reduction

## Discussion

In this study, we enrolled a small sample of THA patients to explore better visualizing the periprosthetic vasculature and to investigate the optimal parameter for balancing metal artifact reduction and vascular visualization in dual-energy CT. We verified VMI combined with O-MAR using appropriate parameters can successfully both reduce metal artifacts and improve vascular visualization. Try our best, we have not retrieved the previously published study focused on the relationship between metal artifact reduction and periprosthetic vascular visualization in total joint replacement and CTA of the lower extremities patients.

Many investigations have demonstrated using O-MAR and VMI can significantly reduce metal artifacts, and the combination could be more effective [17, 18, 26, 28–32]. This was substantiated by the results of our study, which showed significant differences between VMI_MAR_ and CI regarding the attenuation, the width of the hyperdense artifacts as well as the score of artifacts in the subjective analysis. These metrics displayed that the artifacts gradually decreased with increasing VMI settings, which was consistent with previous studies [28, 32]. Hyperdense artifacts were improved significantly as high keV levels primarily reduce beam hardening because of the physical characteristics of the virtual monoenergetic x-rays [26, 31, 33]. Our study further found the downtrend of attenuation of hyperdense artifacts was comparatively sharp at 50–70 keV and relatively flat at 80–120 keV. However, to better visualize the vasculature, low keV VMI was recommended as the preferred parameter for obtaining superior image quality by previous studies [13–16]. A reduction in mean photon energy results in a significant increase in the incidence of photoelectric interactions, particularly as these are increased at and near the k-edge of iodine (33 keV) contained in contrast media. In turn, these increased photoelectric interactions result in increased CT attenuation of vascular-iodinated contrast material [34]. So there seems to be some contradiction in the choice of parameters for the pursuit of minimum artifact and optimal vascular visualization according to current study results. In our study, we chose the highest keV level 120 keV instead of 200 keV, which was recommended as the optimal keV reported previously and not appropriate for this study. With increasing VMI settings, vascular attenuation measured from VMI gradually decreased due to away from the k-edge of iodine (33 keV) [13, 21, 34]. This observation was consistent with data from previous studies [13, 35]. Sufficient attenuation was known to be greater than 180 HU for the pulmonary artery and greater than 200 HU for the aorta [24, 36]. In this study, we defined to be greater than 200 HU as sufficient opacification for the periprosthetic external iliac and common femoral artery which was achieved on VMI_MAR_ at 50–90 keV. Bae et al. reported that sufficient opacification (> 180 HU) was achieved at 40–70 keV for the pulmonary artery [35]. While our study needed to consider the influence of both vasculature and artifacts on image quality. The HU values decreased on the VMI of 80–120 keV in comparison to the conventional image. And the HU values of all structures are lower in high keV images when compared to low keV images and conventional images due to the high keV. However, as the CT value of low keV levels and conventional images increased, the image noise and width of artifacts also amplified which inevitably affected the evaluation of adjacent vessels around the prosthesis, and resulted in a worsening of total imaging quality. Although the artery showed higher attenuation on low keV, it would also result in vascular noise growth. The vascular noise on VMI_MAR_ at 50–70 keV was even higher than that on CI in this study.

**Fig. 4:**
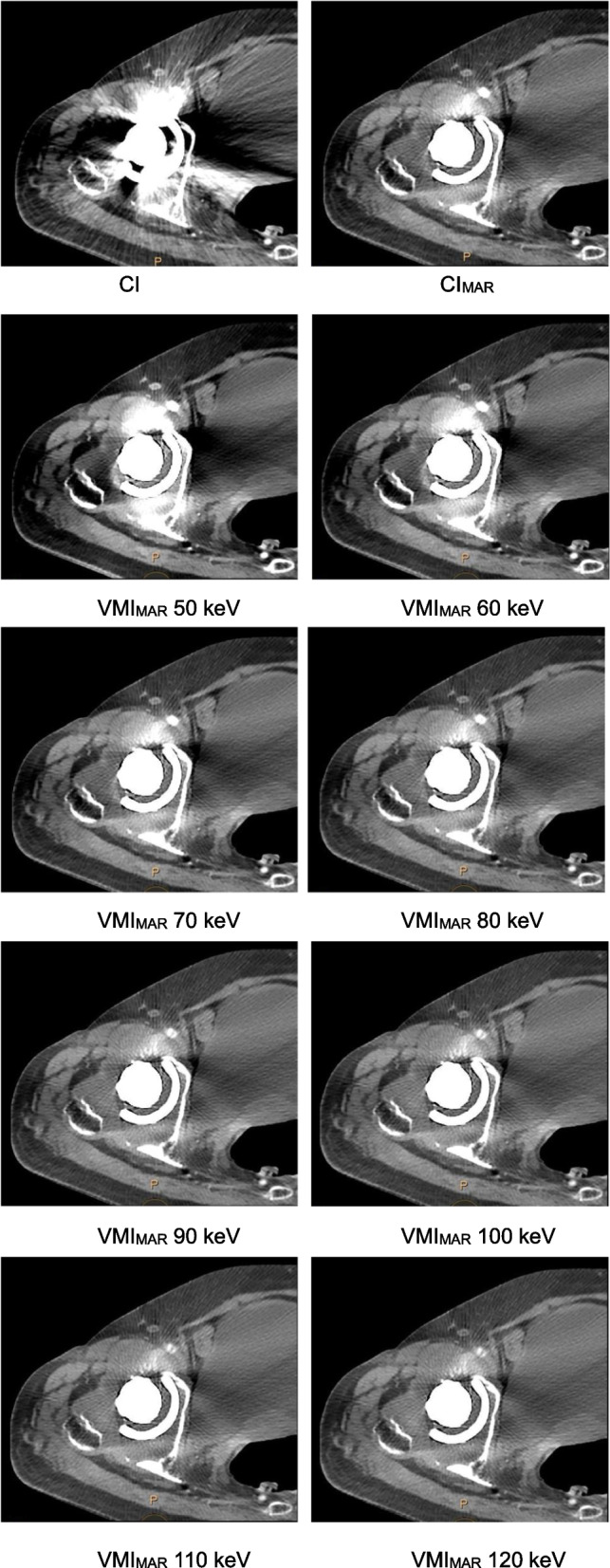
A series of axial enhanced CT images of a patient with THA. Images were reconstructed as CI, CI_MAR_, and VMI_MAR_ at 50–120 keV with 10 intervals (window center 60 HU; window width 360 HU). Images show the metal artifact is gradually reducing with increasing energy level; however, the vascular visualization is worsening. The vascular visualization is terrible above 100 keV, while the vessel is disturbed by artifacts below 70 keV. The best performance is found at 80 keV

**Fig. 5:**
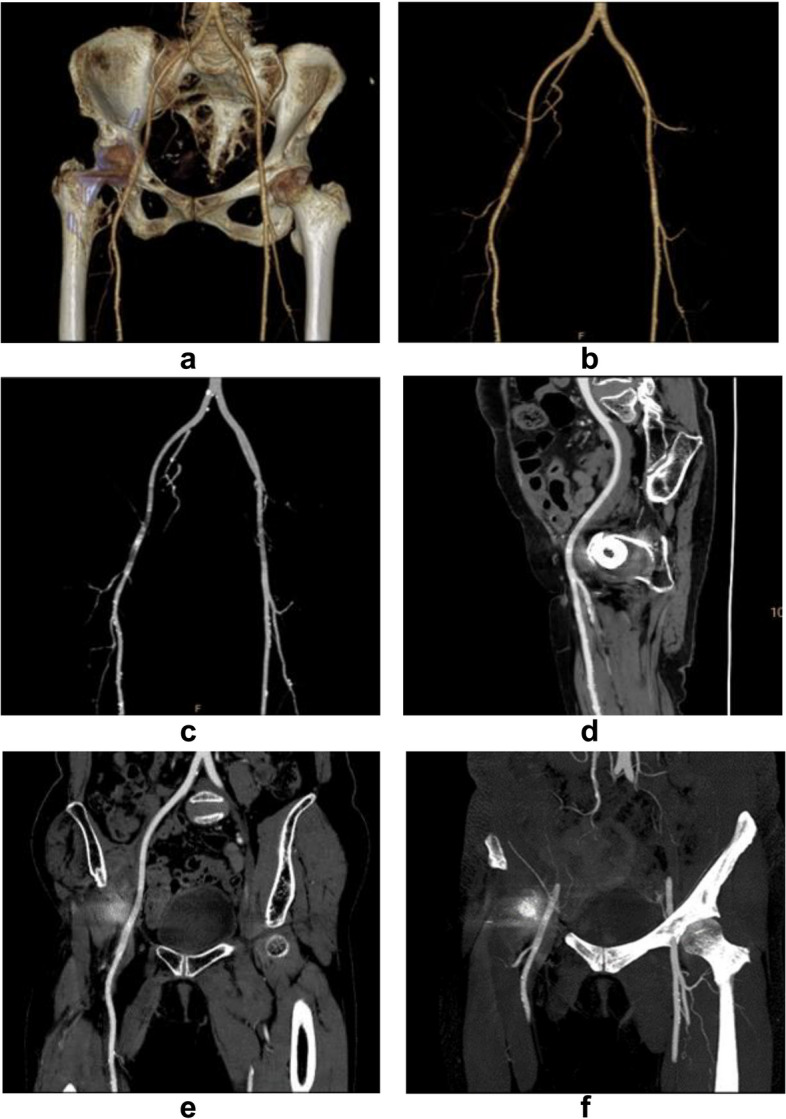
A series of post-processing CTA images of the same patient. 3D volume rendering (VR) (**a**), 3D VR without bone (**b**), maximum intensity projection (MIP) without bone (**c**), curved plannar reconstruction (CPR) (**d**, **e**), and slab MIP (**f**) images of the same patient on VMI_MAR_ at 80 keV. Images show most artifacts are removed and the vessel is slightly affected with acceptable vascular attenuation and sharp vascular contour

We found that following the attenuation and width of metal artifacts gradually decreased with increasing VMI settings, while the periprosthetic vascular attenuation worsened to below 200 HU. Similarly, the score of artifacts gradually improved as the increased keV level, while the score of overall vascular contour and diagnostic evaluation gradually increased at 50 – 80 keV and then decreased at 90–120 keV. This may be due to the impact of the artifact on the vessel being more obvious at low energy levels. Thus, we considered that 80 keV might be an acceptable energy level and equilibrium point to better visualize the periprosthetic artery and reduce metal artifacts (Fig. 4). In this level, the score of overall vascular contour and diagnostic evaluation increased to the top, and the score of artifacts showed most artifacts were removed (Fig. 5). Besides, the vascular attenuation was still over 200 HU and got a satisfied diagnostic confidence. In a study about stent evaluation in lower extremity DE-CTA, Mangold et al. reported 80 keV-VMI + improved image quality, stent visualization, and diagnostic confidence for stent evaluation [37]. In another study about peripheral artery stents, Zhang et al. demonstrated the optimal keV level for peripheral artery stents was concluded at 90 keV [38].

The benefits of O-MAR technique in metal artifact reduction have been reported [17, 18, 39, 40]. In our study, CI_MAR_ also provides relatively satisfactory both reduced metal artifacts and improved vascular visualization. Previous studies have found there were similar absolute attenuation and quality between the degree of enhancement and metal artifact reduction comparing 70-keV VMI to conventional 120-kVp CT [41, 42]. Compared with conventional polychromatic images, VMI has lower noise, higher contrast-to-noise ratio, and can reduce beam hardening artifacts and improve the accuracy of CT numbers [43, 44]. In this study, VMI_MAR_ 80 keV was superior to CI_MAR_ in terms of metal artifact reduction and vascular visualization, which was consistent with previous studies [43, 44].

This study has several limitations. First, the study population and number of THA are relatively small and our results should be confirmed in larger patient populations. Second, quantitative measurements and subjective assessment were all operated manually on the preselected axial images, and the vessels were probably disturbed by the metal artifact. This may confer a certain degree of measurement error. Third, the results only apply to the same CT scanner’s hardware and software configuration. It still needs to further verify if the recommended parameter in our study is also suitable for other devices.

In summary, our results demonstrate that VMI combined with O-MAR using appropriate parameters can provide both reduced metal artifacts and improved vascular visualization. 80 keV on VMI_MAR_ can be an optimal energy level and equilibrium point to give both considerations and is recommended for routine use in the assessment of periprosthetic vasculature of THA patients and CTA of lower extremities in patients with THA.

## Data Availability

The datasets used and/or analyzed during the current study are available from the corresponding author on reasonable request.

## Abbreviations

CI: Conventional image
CI_MAR_: Conventional image combined with orthopedic metal artifact reduction
CTA: Computed tomographic angiography
DECT: Dual-energy computed tomography
HU: Hounsfield Unit
keV: Kiloelectron volt
O-MAR: Orthopedic metal artifact reduction
PAD: Peripheral arterial disease
ROI: Region of interest
THA: Total hip arthroplasty
VMI: Virtual monoenergetic image
VMI_MAR_: Virtual monoenergetic image combined with orthopedic metal artifact reduction
